# The Impact of Foot Orthoses and Exercises on Pain and Navicular Drop for Adult Flatfoot: A Network Meta-Analysis

**DOI:** 10.3390/ijerph18158063

**Published:** 2021-07-29

**Authors:** Ngoc-Tuyet-Trinh Hoang, Shuya Chen, Li-Wei Chou

**Affiliations:** 1 Department of Physical Therapy, Graduate Institute of Rehabilitation Science, China Medical University, Taichung 406040, Taiwan; hngtuyettrinh@gmail.com (N.-T.-T.H.); sychen@mail.cmu.edu.tw (S.C.); 2 Department Physiotherapy, Hong Bang International University, Ho Chi Minh City 700000, Vietnam; 3 Department of Physical Medicine and Rehabilitation, China Medical University Hospital, Taichung 404332, Taiwan; 4 Department of Physical Medicine and Rehabilitation, Asia University Hospital, Asia University, Taichung 413505, Taiwan

**Keywords:** adult flatfoot, exercises, foot orthoses, insoles, orthoses, pain, navicular drop

## Abstract

Background: Adult flatfoot leads to injury and decreased quality of life. The most widely applied noninvasive approaches are wearing foot orthoses or exercising. Both interventions raise controversy about reducing pain and neutralizing foot posture. This study investigated the impact of foot orthoses and exercise on pain and navicular drop (present for foot posture). Methods: Four databases were used: MEDLINE, PubMed, Web of Science, and Cochrane, from the earliest records to November 2020. Randomized controlled studies focused on adult flatfoot that evaluated the effect of exercise and foot orthoses on pain and navicular drop were extracted. We used data analysis to estimate the relative effect of heterogeneity using *I*^2^ and publication bias using funnel plots. Results: Ten studies were identified through to November 2020. Active interventions (AIs) were exercise and exercise combined with foot orthoses; passive interventions (PIs) were foot orthoses and added stretching. Both AIs and PIs decreased pain significantly (SMD −0.94, 95% CI −1.35, −0.54 and SMD −1.4, 95% CI −1.87, −0.92). The AIs reduced pain level better than PIs. Controversially, no treatment was found to affect navicular drop. Conclusion: Both exercise and foot orthoses can reduce pain but not realign foot posture. Exercise alone or combined with foot orthoses showed a better effect on adult flatfoot than only wearing foot orthoses. Active intervention was shown to have better efficacy in reducing pain than passive intervention.

## 1. Introduction

Adult flatfoot can be due to musculoskeletal dysfunction, obesity, diabetes, or rheumatoid arthritis [1]. Flatfoot leads to a high risk of injury, patellofemoral syndrome, lower back pain, and poor quality of life [2,3,4]. Flatfoot was classified into two types, the rigidity, and flexibility of the flatfoot [5]. Nonoperative treatment should be considered for both rigid and flexible flatfoot [6,7]. The treatment principles in the early stages were similar for both conditions, including foot orthoses and some accommodative devices [6,7]. In a study by Fernandez et al. [8], among 835 Spanish adults, 26.5% were found to have flatfoot. Prevalence increased with age. Some individuals may not require any intervention, but management should be recommended when pain and functional problems exist [1]. Methods of flatfoot management include arch taping, insoles, shoes, wedges, braces, exercise, and surgery [2]. Surgery can be used for congenital flatfoot in mild or severe stages, but in one study 4% of patients still reported pain due to ligament laxity [9]. Exercises and foot orthoses are commonly used because of their convenience and economic benefits [10]. Half of all adults with flatfoot in Australia use foot orthoses to prevent excessive foot pronation [11]. The effect of orthoses on adult flatfoot in reducing pain cannot be denied, especially at the early stage of deformity [12,13]. Exercises for flatfoot are widely used and provide some benefits in terms of decreasing pain [14]. Exercises are also effective at strengthening foot muscles forming an arch. Two primary treatments have been designed, which are active and passive intervention. The voluntary muscles produce active interventions (AI), and wearing foot orthoses while doing exercises is also considered as active. For example, active interventions were to practice exercises alone or both do exercises and use foot orthoses. Passive interventions (PI) do not require body effort and involuntary participants during treatment, such as foot orthoses and stretching at the same procedure. The two main approaches are active intervention (AI) and passive intervention (PI) with exercise and foot orthoses. At present, the overall effects of active and passive intervention on adult flatfoot are still unclear because the results depend on skeletal and muscle maturity and the individual’s awareness. Furthermore, flatfoot progresses silently without any acute syndrome of pain or functional impairment. Thus, noninvasive methods that can be used over the long term and are cost-effective could be adopted. The purpose of this study was to analyze the difference in effectiveness between AI and PI in alleviating pain and navicular drop in adult flatfoot.

## 2. Materials and Methods

A comprehensive search was conducted on Web of Science, PubMed, Cochrane, and MEDLINE from the earliest records to November 2020. The two main keywords were “flatfoot” and “interventions” (such as exercises and foot orthoses). The synonym keywords were linked together by the “OR” operator for keywords within one factor, and the “AND” operator for keywords between two elements. The first keywords were “flatfoot,” “pronated foot,” “pronated feet,” “pes planus,” “arch collapse,” “planovalgus,” “flat-arched feet,” “pes plano-valgus,” “rearfoot eversion,” “low arched feet,” and “excessive calcaneal eversion.” The intervention keywords were “short-foot exercises,” “intrinsic foot strengthening and exercises,” “wedge,” “wedged,” “insole,” “insoles,” “anti-pronated shoes,” “orthosis,” and “orthoses.”

A Cochrane PICO (participants, interventions, comparisons, outcomes, and study design) search (2015) was used to collect the appropriate papers.

### 2.1. Participants

The target population was adult patients (older than 18 years) who suffered from flatfoot. Studies were excluded if one group did not have a diagnosis of flatfoot or underwent treatment in the previous 6 months, or had a previous lower limb fracture. Also excluded were studies on patients with flatfoot as a secondary pathology associated with stroke, diabetes, or rheumatoid arthritis.

### 2.2. Interventions

The two noninvasive interventions of interest were exercise and foot orthoses. The specific types of foot orthoses were insoles and orthoses. The interventions were divided into 2 groups: active intervention (exercise alone or combined with foot orthoses) and passive intervention (foot orthoses alone or combined with stretching). Studies comparing groups using other methods (e.g., ultrasound, electric stimulation) were excluded.

### 2.3. Comparisons

Three comparisons were made between groups or with the control group: (1) AI vs. PI; (2) AI vs. control; and (3) PI vs. control. The control groups were sham, non-intervention, and standard intervention, which have a limited kinesiology effect on correcting flatfoot.

### 2.4. Outcomes

The primary outcome was pain as a consequence of flatfoot, which was assessed using a visual analog scale (VAS) and the foot function index [15]. The secondary outcome was navicular drop (ND), which can be visually assessed for improvement quickly and cost-effectively. Studies that used other outcomes were excluded from the research.

### 2.5. Study Design

The studies that were included were randomized controlled trials (RTCs) with at least two comparison groups. There was no language restriction for inclusion. Other research designs were not analyzed.

The extraction of data was based on study design, participant characteristics, intervention groups, assessment tools, and results. The data considered were first author, publication year, population age, number of participants in each group, and intensive intervention. The information used for extraction was the number of participants, and the mean and standard deviation of each group. If the data were not published, we contacted the primary authors for more specific information; if none was available, the study was excluded. The quality of studies was verified by using the physiotherapy evidence database [12] tool. PEDro has 11 items, with “yes” scoring 1 and “no” scoring 0, ranked from 0 to 10, excluding the first item. The 11 items are eligibility criteria, random allocation, concealed allocation, baseline comparability, blinded participants, blinded therapists, blinded assessors, adequate follow-up, intent-to-treat analysis, between-group comparisons, point estimates, and variability. The quality of studies was in the range of poor (score < 4), fair (4–5), good (6–8), and excellent (>9). Two researchers rated the studies independently. If there was any disagreement, a meeting was held to determine the final decision.

R version 3.6.2 software (2019) was used to conduct the network meta-analysis. Both outcomes (pain and navicular drop) were analyzed based on sample size, mean after the intervention in each study, and standardized mean difference (SMD) with a 95% confidence interval (CI). If the study did not report the data after treatment, the data transferred from changed scores to post-test. Heterogeneity was shown by the *I*^2^ statistic and *p*-value of Egger’s test to calculate the study’s inconsistency. In case there were more than 10 studies on an outcome, Egger’s test was used to evaluate the publication bias. Each outcome’s SMD was visualized using a forest plot based on mean difference and 95% CI. Then, R software was used to construct a funnel plot graph to detect the publication bias of all included studies visually. There were two variations that affected research results: errors within studies (such as age, previous injury, and general health) and between studies (sample size, follow-up time). Therefore, the random effect model was the criterion for analysis. The heterogeneity scale, as represented by *I*^2^, is divided into low (<50%), moderate (50%–75%), and high (<75%) ranges. Moderate and high *I*^2^ heterogeneity indicate insufficient combinability caused by significant inconsistency between studies. Thus, with a heterogeneity of research higher than 50% and *p*-value < 0.05, a subgroup was analyzed for ND and pain outcomes to interpret the results. The subgroups were based on similar characteristics between studies for each outcome.

## 3. Results

### 3.1. Research Properties

#### 3.1.1. Eligible Studies

All 775 studies were added to Endnote X9, and duplications were eliminated, leaving 635 papers. Then 529 studies were excluded because they focused on diseases unrelated to this study, such as internal fixation of the foot bone and musculoskeletal impairment of the lower limbs.

After papers were excluded, the remaining 106 studies were considered, and 93 studies were excluded based on reading the full text. Three more studies were excluded because the pain outcome was assessed by a different index. One index set pain scores in the opposite direction from the VAS (more pain = lower score), and the other evaluated the comfort of treatment. The 10 studies left were divided into 3 main groups based on the different comparisons they made.

**Figure 1:**
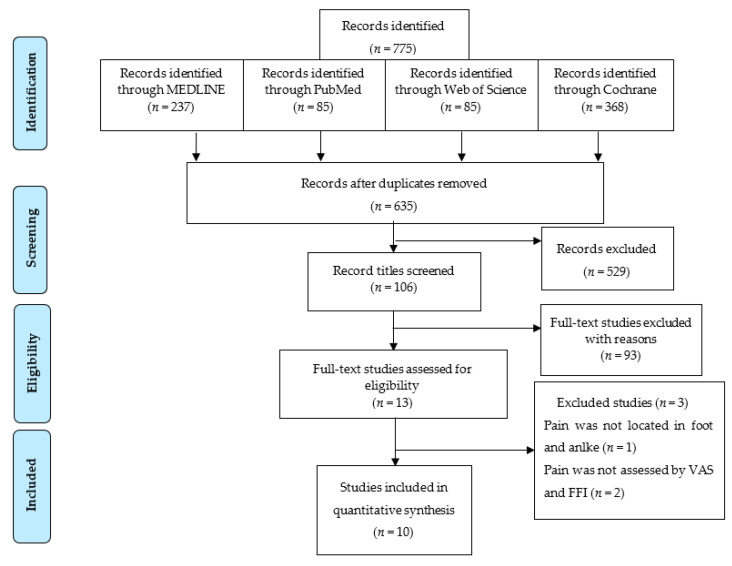
PRISMA flowchart.

The 10 papers were classified into three groups, comparing AI vs. PI, AI vs. control, and PI vs. control. The entire procedure followed the Preferred Reporting Items for Systematic Review and Meta-Analyses (PRISMA) diagram (2009), shown in Figure 1. Three studies, by Andreasen et al. [16], Yurt et al. [17], and Kulig et al. [18], designed experimental groups including standard intervention, insole group, exercise group, and mixed insole and exercise group. Therefore, we divided the research data into several pairwise comparisons to meet the criteria of AI vs. PI, AI vs. control, and PI vs. control. The comparison in the study by Andreasen et al. [16] was arranged into Andreasen (1), (2), (3), (4), and (5) to compare the data for exercise vs. foot orthoses, exercise vs. control, foot orthoses vs. control, foot orthoses and exercises vs. control, and foot orthoses and exercise vs. foot orthoses, respectively. The comparison in the study by Yurt et al. [17] was arranged into Yurt (1) to compare the data between CAD-CAM foot orthoses vs. control and Yurt (2) for conventional foot orthoses vs. control. The comparison in the study by Kulig et al. was arranged into Kulig (1) to compare the data for concentric exercise vs. foot orthoses and Kulig (2) for eccentric exercise vs. foot orthoses.

#### 3.1.2. Trials and Study Characteristics

Ten studies enrolled 385 participants, with a median sample size of 38.5 participants. Among the participants, 50% had an average body mass index (BMI) (18.5–24.9), 30% were overweight (25–29.9), 9% were obese (>30), and 11% were not reported. The overweight and obese participants were mostly in the studies by Houck et al., Andreasen et al., and Kulig et al. Their ages ranged from early adult (20–40 years old) to middle age (40–65 years old). Overall, the studies included recruited both male and female candidates, divided into 221 young adults (57%) and 164 middle-age adults (43%). All of the participants were from universities, outpatient hospitals, and athletic centers. The participants’ characteristics are listed in Table 1.

The information listed in Table 2 represents the analyzed treatment effect from 2 to 16 weeks only for consistent follow-up time. In general, compared to the control group, each study reported pain reduction with both passive and active intervention. Almost all of the studies reported that AI tended to be more useful for releasing pain than PI, except for Houck et al. [19]. In the NDT, all studies showed incoherence and fluctuation effects among AI, PI, and control groups.

#### 3.1.3. Intervention Design

The muscles targeted for strengthening were the toe muscles and intrinsic and extrinsic foot muscles (including quadriceps, gluteus, anterior/posterior tibialis, and gastrocnemius soleus). The most commonly used exercises for muscle strength were short foot exercises, heel raises (unilateral or bilateral), plantar flexion and adduction (strengthening posterior tibialis), and Achilles tendon stretching, as listed in Table 3.

The foot orthoses were made of semi-rigid materials including ethylene-vinyl acetate (EVA) shore 35 A, polypropylene, thermo-molded composite, and rubber-like material. Some studies required the participants to use the foot orthoses frequently, especially during physical activities. Some foot orthoses were made with orthotics, such as computer-aided design/computer-aided manufacturing (CAD-CAM) and custom-made orthoses. Other studies used support arch products from medical instrument companies such as Biomechanical Services and DJO. All foot orthoses supported the medial ankle side, the medial longitudinal arch (MLA), or the rearfoot’s medial side, as listed in Table 4.

**Table 1:** Characteristics of selected participants.

First Author	Number of Participants	Gender	Active Intervention			Passive Intervention			Control		
			Participants	Age	BMI	Participants	Age	BMI	Participants	Age	BMI
Yurt [17]	45	F, M	0	No	No	22	21.73 (2.89)	23.03 (3.48)	23	21.09 (1.95)	23.32 (3.28)
						22	23.05 (5.53)	24.11 (4.15)			
Park [20]	28	F, M	14	24.71 (1.77)	NR	0	No	No	14	23.50 (2.03)	NR
Shih [21]	24	F, M (18 M, 6 F)	0	No	No	12	31.3 (8.3)	22.3 (2.9)	12	34.4 (9.8)	23.1 (2.8)
Andreasen [16]	80	F, M (65 F, 15 M)	20	44 (13)	26.8 (5.9) *	20	41 (16.2)	26.4 (5.6) *	20	43 (11.9)	25.8 (4.33) *
			20	44 (16.2)	27.7 (4.8) *						
Kulig [18]	36	F, M (28 F, 8 M)	12	55.3 (16.4)	32.0 (9.24) *	12	51.3 (17.2)	28.7 (6.26) *	0	No	No
			12	49.4 (12.6)	28.5 (7.09) *						
Houck [19]	36	F, M	19	57 (12)	30 (6)*	17	58 (9)	31 (5) *	0	No	No
Jeong [22]	12	F, M	7	52.57 (16.13)	22.6 (2.37)	0	No	No	5	53.2 (12.61)	24.02 (3.63)
Okamura [23]	20	F, M (17 F, 3M)	10	19.7 (0.9)	19.8 (1.4)	0	No	No	10	20.2 (1.5)	21.1 (2.1)
Pabón-Carrasco [24]	90	F, M	45	19.45 (0.38)	24.13 (4.16)	0	No	No	45	20.92 (1.1)	21.65 (3.35)
Kim [25]	14	F, M	7	24.0 (1.9)	NR	7	24.1 (1.5)	NR	0	No	No

Note: All data are in mean and standard deviation. BMI, body mass index; * overweight or obese (BMI ≥ 25); F, Female; M, Male; NR, Not reported.

**Table 2:** Descriptions of included studies.

No.	First Author, Year Study Design	Participant Recruitment	Active Intervention	Passive Intervention	Control	Assessment Tools	Follow-Up Time	Results
1	Okamura, 2020 RCT	FPI > 6	Short foot exercises	No	Control: no intervention	Foot kinematics: 3D motion analysis, navicular drop during gait, FPI, ultrasound muscle thickness	8 weeks	FPI: inversion/eversion significantly improved; time required for navicular height: minimum value decreased significantly
2	Pabón-Carrasco, 2020 RCT	FPI > 6	Short foot exercises	No	Non-biomechanical function (NBF) exercise	NDT and FPI	4 weeks	No values were found for foot posture between 2 groups; posture was modified in both groups in initial state, and ND value decreased in pain and posture
3	Yurt, 2018 RCT	FPI > 6	No	CAD-CAM	Flat insoles	VAS, foot function index, short form−36	8 weeks	Pain on CAD-CAM, conventional lower than control after 2 months
4	Kim, 2016 RCT	NDT > 10 mm	Short foot exercises	Arch support insoles	No	Navicular height NDT, Y balance tests	5 weeks	NDT: SFE showed significant decrease Y balance: both SFE and insoles showed significant increase
5	Houck, 2015 RCT	PTTD stage II	Orthoses and stretching and isotonic strengthening	Orthoses and stretching	No	FFI (pain) and short musculoskeletal function assessment	6 and 12 weeks	Significantly improved pain and function
6	Andreasen, 2013 RCT	Calcaneal valgus > 6°	Exercise	Insoles	Standard intervention	Pain, static and dynamic foot postures: calcaneal angle, navicular drift, drop, and height	4 and 12 months	Pain reduction during walking; no differences seen between groups at 4 months
			Insole and exercise					
7	Park, 2012 RCT	Footprint	Abductor hallucis, digit. flexor, anterior and posterior tibialis strengthening	No	Control	Foot structure NDT, mass pressure, motion analysis	8 weeks	Foot strengthening exercise is feasible and suitable for individuals with hallux valgus with flexible flatfoot Significantly increased outcomes of structural and plantar foot pressure
8	Shih, 2011 RCT	NDT > 10 mm	No	Custom-made insoles	Control: soft flat insoles	VAS	2 weeks	Pain incidence reduced in treatment group after 2 weeks, pain intensity score decreased after orthosis application
9	Kulig, 2009 RCT	Arch index PTTD stage I, II	Orthoses and concentric exercise	Custom-made insoles, stretching	No	VAS pain, 5 MWT, FFI	6 and 12 weeks	Pain reduced in all groups, orthoses eccentric most improved, orthoses least improved
			Orthoses and eccentric exercise					
10	Jeong, 2007 RCT	PTTD stage I or IIa	Stretching and strengthening, balance training	No	No exercise	Pain, ROM, muscle, AOFAS, 5 MWT	6 weeks	Reduced pain in exercise group, increased plantar/dorsiflexion

RCT, randomize control trials; NDT, navicular drop test; FPI, foot posture index; PTTD, posterior tibialis tendon dysfunction; ROM, range of motion; MWT, minute walk test; VAS, visual analog scale; FFI, foot function index, CAD-CAM, computer-aided design/computer-aid manufacturing; AOFAS, American Orthopedic Foot & Ankle Society; SFE, short foot exercises.

**Table 3:** Exercise design.

First Author	Design												
	Bilateral Heel Raise	Unilateral Heel Raise	Seated Heel Raise	PF and FAdd	Towel Grasp	Calf Stretching	Shor Foot Exercises	TA Resistance Strengthening	Abductor Halluces Strengthening	Toe Spread	Quadriceps Strengthening	Gluteus Strengthening	Number of Exercises
Kulig [18]	No	No	No	Yes	No	Yes	No	No	No	No	No	No	2
Houck [19]	Yes	Yes	Yes	No	No	Yes	No	No	No	No	No	No	4
Yurt [17]	No	Yes	No	No	Yes	Yes	No	No	No	No	No	No	3
Shih [21]	No	No	No	No	No	No	No	No	No	No	No	No	0
Kim [25]	No	No	No	No	No	No	Yes	No	No	No	No	No	1
Andreasen [16]	Yes	Yes	No	No	Yes	Yes	Yes	No	No	Yes	Yes	Yes	8
Okamura [23]	No	No	No	No	No	No	Yes	No	No	No	No	No	1
Jeong [22]	Week 3–6	Week 5–6	Week 1–6	Week 1–6	No	Yes	No	No	No	No	No	No	5
Pabón-Carrasco [24]	No	No	No	No	No	No	Yes	No	No	No	No	No	1
Park [20]	No	No	5 min	No	5 min	No	No	5 min	5 min	No	No	No	4

PF, plantar flexion; FAdd, foot adduction; TA, anterior tibialis.

**Table 4:** Foot orthoses design.

First Author	Foot Orthoses Design	Foot Orthoses Details and Properties	Posting		Frequency Wearing Foot Orthoses
			Rearfoot	MLA	
Kulig [18]	Orthoses	Biomechanical service, thermo-molded composite, rigid shell	NR	NR	90% hours walking
Houck [19]	AirLift, Aircast	Aircast (DJO Global Inc, Vista, CA, USA) including ankle stirrup and MLA support	NR	NR	9.9 h a day
Yurt [17]	CAD-CAM or conventional insoles	35 shore A main insoles 3 mm thick, and 15 shore EVA for covering; 4 to 6 mm metatarsal pad	6° medial heel wedge	8–12 mm MLA	Frequent use, especially for outdoor activities
Shih [21]	Semi-rigid rearfoot medial wedge	2 mm Poron (rubber-like)	Off-the-shelf 5° EVA	Wedge 6–8 mm from longitudinal midline to medial edge	While running on treadmill
Kim [25]	Custom-made orthoses	Thermoplastic (3.2 mm thick Aquaplast-T)	NR	MLA with shore 20°, height at least 15 mm with medial arch support	30 min 3/week, 5 weeks
Andreasen [16]	Custom-made insoles	EVA shore A 35 made by orthotic	NR	NR	2–8 h/day, compliance not monitored

EVA, ethylene vinyl acetate; CAD-CAM, computer-aided design/computer-aided manufacturing; MLA, medial longitudinal arch; TA, tibialis anterior; NR, not reported.

### 3.2. Data Analysis

#### 3.2.1. Study Synthesis

**Figure 2:**
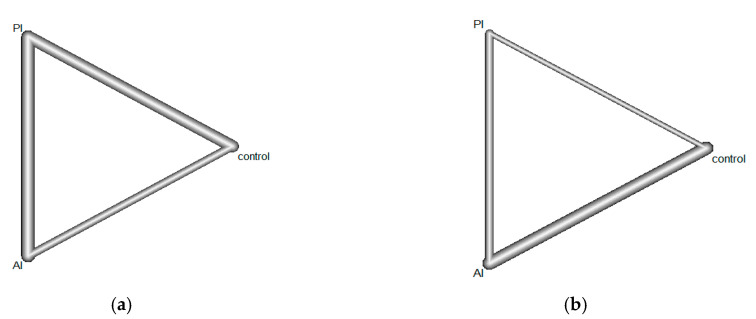
Net graphs of included studies. AI, active intervention; PI, passive intervention. (**a**) Net graph of pain; line thickness indicates number of studies with AI vs. PI (*n* = 5), AI vs. control (*n* = 3), and PI vs. control (*n* = 4). (**b**) Net graph of navicular drop; line thickness indicates number of studies with AI vs. PI (*n* = 3), AI vs. control (*n* = 5), and PI vs. control (*n* = 1).

The network graphs represent the connection of the three comparisons for one outcome (Figure 2). The line thickness in the graph indicates the number of studies. The VAS pain score was 5 for AI vs. PI and 3 for AI vs. control, and five studies compared PI vs. control. On the static navicular drop test, there were 3 studies for AI vs. PI, 5 studies for AI vs. control, and 1 study for PI vs. control group. All data were imported into R software, and direct and indirect comparisons were made with the random effect model. Control group was considered as one of the treatments. Therefore, there were three treatments, AI, PI, and control. The direct comparison was performed to analyze the immediate difference in AI vs. PI, AI vs. control, and PI vs. control. The indirect comparison indirectly distinguished the differences between treatments by considering the “third party”; for example, AI vs. PI had two indirect comparisons as the third party: AI vs. control and PI vs. control. Therefore, both direct and indirect evidence (compared with the control group) estimated the effect of AI and PI on adult flatfoot.

#### 3.2.2. Forest Plot of the Netsplit of Each Outcome

For visualization, the results were displayed in a forest plot of the netsplit. Each row shows the path-based effect estimate. The direct evidence in the network estimate has the main role of both the network structure and the precision. The direct evidence was the clue to identify the result’s influence, which means the more direct the evidence, the greater its impact on the comparison. Direct and indirect estimates were synthesized in a diamond shape in the network comparison. Table 5 lists the study contributions for comparison.

**Figure 3:**
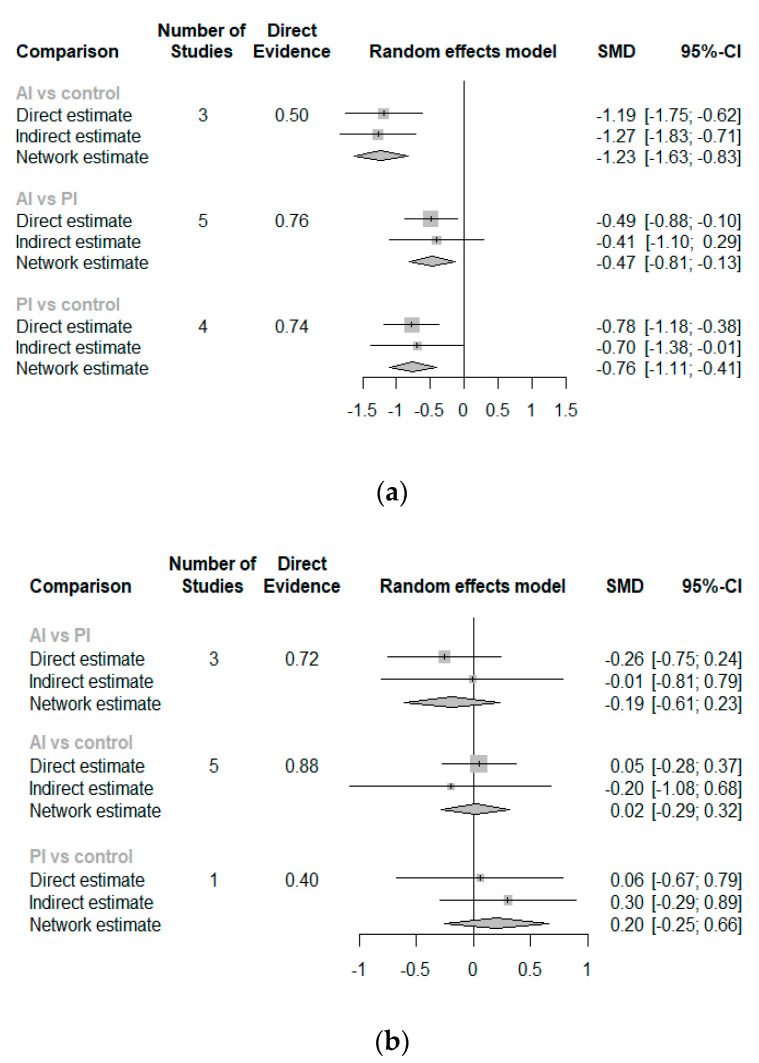
Forest plot of (**a**) pain and (**b**) navicular drop.

In terms of reducing pain from flatfoot, AI was more likely to reduce pain than PI, (SMD −0.47, 95% CI −0.81, −0.13). Additionally, both AI and PI were shown to have an effect on flatfoot compared to controls (SMD −1.23, 95% CI −1.63, −0.83; SMD −0.76, 95% CI −1.11, −0.41, respectively) (Figure 3a). On the contrary, neither AI or PI showed a superior effect on the ND test (SMD −0.19, 95% CI −0.61, 0.23). Similarly, after AI and PI, foot alignment (assessed by ND test) did not improve compared to control (SMD 0.02, 95% CI −0.29, 0.32; SMD 0.20, 95% CI −0.25, 0.66, respectively) (Figure 3b).

#### 3.2.3. Treatment Effect Ranking

To answer the question of which intervention was better, we analyzed the probability rank (P-score). The P-score, based on point estimates and standard errors of NMA, can explain a treatment being better than competitors. The P-score of each treatment showed its ranking among the others; the most effective P-score is 1 and the worst is 0, and a higher score indicates a better effect [26]. In terms of alleviating pain, AI (0.99) demonstrated superior effect and roughly double the impact compared to PI (0.5), while the control group had no change in pain. However, based solely on ranking, we could not directly determine each treatment’s effect on the disease. Adding other tools, such as a netsplit plot, helped in visualizing the overall picture. In terms of navicular drop, AI showed a similar impact to the control or conventional intervention (P-score 0.67 and 0.63). Interestingly, AI was shown to have more than 3 times the efficacy of PI in foot realignment (0.67 and 0.19, respectively). However, they still could not reconstruct the foot into a neutral position (as shown on the netsplit forest plot, Figure 3b).

### 3.3. The Consistency between Evidences

In this NMA, there were 3 pair-wise comparisons, which were AI vs. PI, AI vs. control, PI vs. control. Direct and indirect evidence of each pair-wise comparison contributed to the result network evidence. There was some inconsistency (within designs) that might perturb the network estimates effect, which came from the direct and indirect estimate effect, such as differences in the participants’ characteristics between each group. The network’s consistency was checked by splitting each pair-wise comparison into direct and indirect evidence estimates. If the *p*-value was less than 0.05, it represented significant disagreement between direct and indirect estimation (so-called inconsistency). As listed in Table 6, the *p*-value between a direct and indirect estimate in both pain and NDT outcome was much more than 0.05 (0.84 and 0.61, respectively). These *p*-values mean the significant consistency between the direct and indirect estimates in the random-effect model. Both direct and indirect evidence could be reliably evaluated to show pain and ND outcomes.

### 3.4. Heterogeneity among Included Studies

Differences in clinical variation led to heterogeneity. The intervention design or the population characteristics of each study inevitably affected the reliability between studies. For example, the population target might be different, from young adults to old adults, or the follow-up time may be inconsistent. Different studies could lead to different results, which was acceptable for some criteria. Hence, heterogeneity was represented as a percentage that shows whether these differences could be acceptable; lower than 50% would mean low heterogeneity, which could be acceptable. In the results, *I*^2^ = 27.3% with *p*-value = 0.18 for pain and *I*^2^ = 19.8% with *p*-value = 0.27 for NDT. These indicated that there was low heterogeneity between pain and navicular drop outcomes.

### 3.5. The Impact of Combination of Exercises and Foot Orthoses Versus Foot Orthoses Alone in Pain on 12 to 16 Weeks Follow-Up

Furthermore, the original result has already displayed the overall effect of AI (exercises alone, or exercises combine with foot orthoses) and PI (foot orthoses). The question was whether there was any difference between foot orthoses alone and the combination of foot orthoses with exercises on adult flatfoot. Hence, this second subgroup used the meta-analysis to answer.

**Figure 4:**
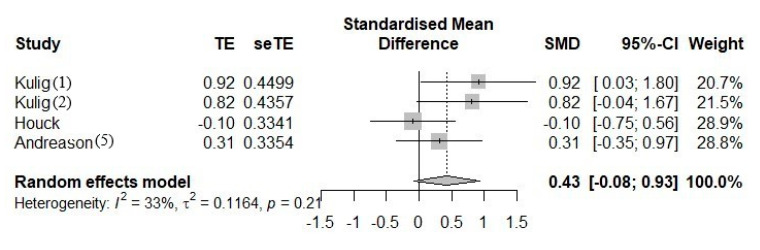
The forest plot between mixing foot orthoses with exercises and foot orthoses in pain.

The Figure 4 forest plot was used to investigate the standardized mean difference (SMD) (95% CI) and the weight of every single research in a random-effects model. In general, the figure showed that most instances of exercises and foot orthoses used together was more likely to reduce pain than the single-used foot orthoses (SMD [0.43], 95% CI [−0.08; 0.93]). However, this difference was inappreciable because the result crosses the midline. Additionally, the heterogeneity *I*^2^ = 33% (less than 50%) with *p*-value = 0.21 proved low heterogeneity or coherence altogether.

### 3.6. Risk of Bias and Publication Bias

In the evaluation of evidence there was a risk of bias. PEDro was used to assess the risk of direct evidence bias in terms of internal validity and statistical information. The studies were evaluated by two independent researchers and no disagreements occurred. As shown in Table 7, four studies (Houck et al. [20], Kulig et al. [18], Kim et al. [25], and Park et al. [20]) were assessed as fair quality, and six studies (Jeong et al. [22], Andreasen et al. [16], Pabón-Carrasco et al. [24], Okamura et al. [23], Yurt et al. [17], and Shih et al. [21]) were of good quality. The study limitations were evaluated in these 10 studies, to ensure that the contribution of the source was reasonable.

Among the included studies that were randomized controlled trials, seven were of good quality, and four were of fair quality. There were two criteria that almost all of the studies did not meet: blinded participants and blinded therapists. This mean the participants did not know the participants’ group allocation and were unable to distinguish differences in treatment. Most studies could not meet these criteria because exercising was a voluntary action, which required the awareness of participants and therapists.

**Figure 5:**
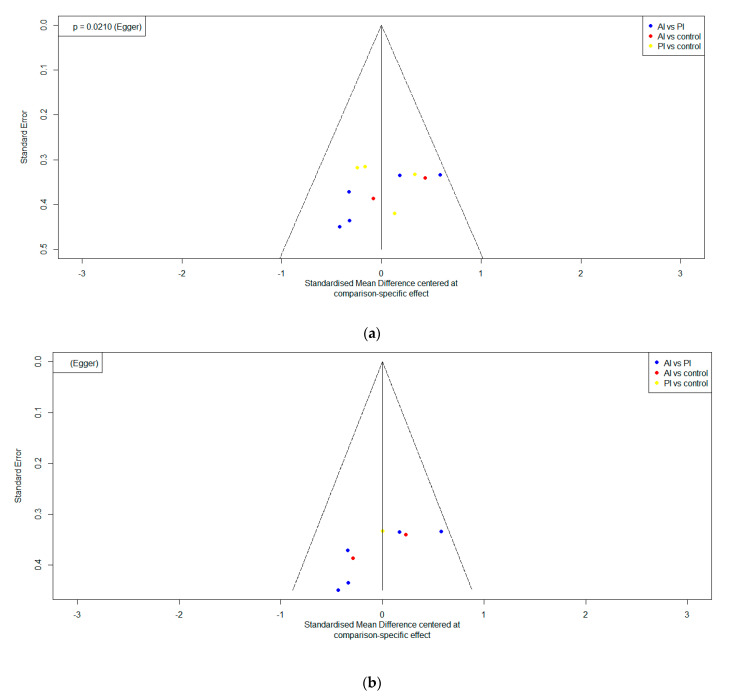
Funnel plot of publication bias of (**a**) pain and (**b**) navicular drop.

The studies were chosen carefully; some publication bias led to overestimation. The funnel plot and Egger’s test detected potential impact bias. The vertical axis indicates the standard error funnel plot approximated by the sample size and effect estimate, and the horizontal axis indicates the standardized mean deviation. In both outcomes, the studies were asymmetrically distributed (Figure 5a,b). The marginal publication bias was presented for the pain score group with an Egger’s test score of 0.021 (lower than <0.05) (Figure 5a) means the unsubstantial asymmetrical of funnel plot in pain. The Egger’s test detected that there was no significant difference between large and small studies (Figure 5a,b).

## 4. Discussion

This study was to show the effectiveness between AI and PI in alleviating pain and navicular drop in adult flatfoot. Both exercise and foot orthoses could relieve pain but were unsuccessful at changing navicular drop during a maximum 16-week follow-up. Active interventions such as exercise alone and exercise combined with foot orthoses achieved effects on the alleviation of pain that were twice as effective compared to passive treatment. This result suggested that exercise and foot orthoses can reallocate foot pressure but cannot realign foot structure.

First, navicular drop was not significantly decreased after using AI or PI over 2 to 16 weeks of follow-up. This result was similar to previous studies [12,13]. Banwell et al. [13] conducted a systematic review of 13 studies on the effect of orthoses on flexible flatfoot. The authors concluded that there was a decreased pain effect, but limited evidence for increased rearfoot eversion. Furthermore, the foot arch was supported passively by bones and ligaments and actively by intrinsic and extrinsic foot muscles [27]. Exercise was only found to manipulate the active components (foot muscles) but is unable to control the passive components (bones and ligaments) of the foot arch. Even the combination of exercise and foot orthoses did not show a significant impact on flatfoot realignment in an adult population. The bony foot arch changed during the early childhood and became mostly stable at 7 years old [28]. Hence, the effects of exercise and foot orthoses could change over time, with this treatment being more effective in childhood and gradually less so in adulthood.

In this study, exercise and foot orthoses showed limited results in foot arch construction. However, the foot structure conservation was undeniable during foot orthoses and exercises interventions. Some adult-acquired flatfoot deformity is from high-demand activities, such those performed by athletes and soldiers, or injuries that cause a collapsed arch, such as tibial posterior tendon dysfunction [11,18,21]. Both foot orthoses and exercises maintained the over-pronated foot, which did not become worse even though the arch morphology was already fixed.

Second, both exercise and foot orthoses successfully reduced pain from adult flatfoot over about 16 weeks of follow-up. Both active and passive interventions showed better effects than the control, and AI showed a double impact compared to PI in terms of pain relief. Flatfoot caused pain by exertion during activities and reduced by relaxation in the early stage [29]. An excessively pronated foot enhanced stress on joint surfaces, spring and Achilles ligaments, and other structures [30]. This mechanism caused pain, which can inhibit muscles and result in atrophy, and then limited movement [31]. Strengthening could stabilize the muscles and enhance proprioceptive feedback [27]. Exercise also supported movement to prevent chronic overload on metatarsals, stress fractures, and stress reaction [27,31]. The combination of exercise and foot orthoses could release the tension and stabilize the surrounding soft tissue [27]. Therefore, active intervention can control the pain coming from the joint moving in extreme ranges [27]. The research of Megan et al. [14] had similar results. The authors conducted a systematic review of three studies on posterior tibial tendon dysfunction (which causes adult-acquired flatfoot deformity) with exercise. They found that pain was alleviated when participants applied specific exercises and orthoses.

The foot orthoses materials should also be considered. Semi-rigid materials such as EVA, poron, and polypropylene might be better than rigid ones [32,33]. Rigid materials can exacerbate the release syndrome. Semi-rigid insoles act as artificial foot arches that can absorb loads for normal foot pronation instead of no pronation at all, unlike rigid ones [34]. The semi-rigidity of the insoles was the reason that foot orthoses could reduce the pain from a collapsed foot. The dispersion forced evenly along the longitudinal arch could release the tension of medial pressure [32]. Hence, foot orthoses can decrease pain and provide comfort for the flatfoot ligaments during daily life activities.

The orthoses designed for navicular drop were not consistent throughout the studies. Some foot orthoses were designed to correct overpronated rearfoot by inserting the wedge on the medial calcaneus, and some foot orthoses were intended to lift the longitudinal arch from 8 to 15 mm off the ground. All authors had a similar concept but different orthoses designs, which was the confounding factor. Flatfoot came from a misalignment of the ankle valgus, pronated rearfoot, supinated and abducted forefoot, and then unstable medial longitudinal. The primary purpose of foot orthoses was to lead the bone structure to a neutral position and support muscles during regular activity.

Third, the participants’ characteristics were factors that had an effect on adult flatfoot. As we can see in Table 2, the participants included in the subgrouped studies (Andreasen, Kulig, and Houck) had body mass index values ranging from overweight to obese (BMI > 25) and their ages were in the older adult range (>40 years). The significant results of the treatments support the observations that older age and overweight make flatfoot more serious [4].

According to Xu Tao et al. [35], research in network meta-analysis indicated that surgical treatment of adult flatfoot could improve symptoms and foot alignment over more than a year (12 to 42 months of follow-up). The recruitment criterion was moderate to severe flatfoot deformity. The post-operative maintenance programs were not mentioned in this study. Xu Tao et al.’s study raised a question regarding what the appropriate treatment should be for the early stage of mild or moderate adult flatfoot, and the maintenance programs after surgery might need more research in the future. Older age went along with additional risk factors, so surgery for adult flatfoot should be performed with caution and whenever maintenance treatment fails [9]. Furthermore, there was also a correlation between adult flatfoot and obesity, hypertension, and diabetes [29].

The data presented in Figure 4 suggests that doing both treatments together seems to reduce pain better than only wearing foot orthoses. However, this result was confounded because of a negative result from Houck et al. [19], (−0.1 [−0.75; 0.56]). While the other comparisons show positive results, proving the mixed treatment leads to better pain alleviation than footwear, Houck et al. [19] reported a contrary result. The opposite result of Houck et al. changed the overall effect and caused bias while evaluating the effect of foot orthoses and mixing foot orthoses with exercises on adult flatfoot.

The five criteria for recruitment of flatfoot participants were foot posture index (FPI), navicular drop test, stage I and II posterior tibial tendon dysfunction (PTTD), footprint, and calcaneal valgus. Stage I and II PTTD were defined as early inflammation of tendons leading to flexible flatfoot. Similarly, NDT indicated the differences in foot arches between non-weight-bearing and weight-bearing higher than 10 mm, which means flexible flatfoot. In contrast, with FPI score > 6, flattened foot print, and calcaneal valgus > 6 in the standing position indicated collapse of the foot arch, with either flexible or rigid flatfoot. The inconsistent recruitment criteria confounded the results; the interventions could not determine the treatment effect on either flexible flatfoot or rigid flatfoot.

Furthermore, there was no consensus among the time points for assessing pain throughout the studies; some considered pain after doing exercises or daily activity, and some evaluated pain in general (resting, walking with insoles, and walking without insoles). The time points for assessing VAS and FFI in terms of pain and pain location should be considered. Different properties of pain could interfere with the results.

The exercise design in 10 of the studies was mostly focused on strengthening the muscles; to some extent, balance and proprioceptive exercises should be added to prevent injury. Some of the described exercise parameters were ignored, especially the resting time, tension time, recovery, and repeating time. Detailed descriptions were essential clinically and could help in translating exercises from academic to practical settings. Slight changes in parameters lead to big differences in physiological effects which can be targeted to the purpose of recovering the muscle tendons in flatfoot. Furthermore, the exercise designs varied in different studies, and there were various versions of some of the same exercises, such as heel raising. Such dissimilar exercise designs might confuse specialists and confound the overall results.

Another objective factor was participants’ awareness. Active interventions require paying attention to the procedures and repeating them frequently. Participants’ distraction was inevitable, which therapists had difficulty monitoring. Therefore, passive interventions seem to be more popular than active ones [36], even though active interventions show more benefits for flatfoot treatment.

This study had some limitations. The database engines had different designs. The same searching steps were impossible to use throughout all websites. Therefore, all included studies were collected based on similar searching strategies. All included studies were randomized controlled trials, which limited the synthesis of studies with other research designs. Practicing exercises or using foot orthoses were the visually vivid criteria from RCTs (blinded participants and therapists) limited from enhancing data. Other kinds of treatment that were not mentioned might have positive effects on adult flatfoot. With the concept of exercise, the general definition of which confuses users when applying it in reality, it is hard to unify a specific procedure for only adult flatfoot. Additionally, pain and navicular drop outcomes were based mostly on the observations of researchers and the feelings of participants. This leaves the results open to the presence of human bias.

## 5. Conclusions

The effects of the habitual use of foot orthoses and exercise was clearly found to be the alleviation of pain in adult flatfoot. Active interventions were found to be able to reduce pain more effectively than passive intervention. However, both interventions did not change the foot structure in adult flatfoot, which might require intensive treatment such as surgery.

## Figures and Tables

**Table 5:** Contributions of each study in each comparison.

Outcome	Pain			Navicular Drop		
Comparison	AI Vs. PI	AI Vs. Control	PI Vs. Control	AI Vs. PI	AI Vs. Control	PI Vs. Control
Number of direct comparisons	5	3	4	3	5	1
Study contribution	Andreasen [16] (3) Andreasen [16] (5) Kulig [18] (1) Kulig [18] (2) Houck [19]	Andreasen [16] (2) Andreasen [16] (4) Jeong [22]	Andreasen [16] (1) Yurt [17] (1) Yurt [17] (2) Shih [21]	Kim [25] Andreasen [16] (3) Andreasen [16] (5)	Andreasen [16] (2) Andreasen [16] (4) Park [20] Okamura [23] Pabón-Carrasco [24]	Andreasen [16] (1)

**Table 6:** The evidences of each comparison for pain and navicular drop.

Outcomes	Comparison	k	prop	nma	direct	indir	diff	*p*-Value
Pain	AI vs. PI	5	0.76	−0.47	−0.49	−0.41	−0.08	0.84
	AI vs. control	3	0.5	−1.23	−1.19	−1.27	0.08	0.84
	PI vs. control	4	0.74	−0.76	−0.69	−0.70	−0.08	0.84
Navicular drop test	AI vs. control	5	0.72	−0.19	−0.26	−0.01	−0.25	0.61
	AI vs. PI	3	0.88	0.02	0.05	−0.20	0.25	0.61
	PI vs. control	1	0.40	0.20	0.06	0.30	−0.25	0.61

Noted: k, number of studies providing direct evidence; prop, direct evidence proportion; nma, estimated treatment effect based on SMD in network meta-analysis; direct, estimated treatment effect based on SMD derived from direct evidence; indir, estimated treatment effect based SMD derived from indirect evidence; diff, difference between direct and indirect treatment estimates; *p*-value, *p*-value of test for disagreement (direct versus indirect).

**Table 7:** Quality of examined studies by Physiotherapy Evidence Database score.

Study	Criteria												
	1	2	3	4	5	6	7	8	9	10	11	Total	Quality
Houck [19]	1	1	0	1	0	0	0	1	0	1	1	5/10	Fair
Kulig [18]	1	1	0	0	0	0	0	1	0	1	1	4/10	Fair
Jeong [22]	1	1	0	1	0	0	0	1	1	1	1	6/10	Good
Andreasen [16]	1	1	1	1	0	0	1	1	0	1	1	7/10	Good
Kim [25]	1	1	0	1	0	0	0	0	0	1	1	4/10	Fair
Park [20]	1	1	0	0	0	0	1	0	0	1	1	4/10	Fair
Pabón-Carrasco [24]	0	1	0	1	0	0	1	1	1	1	1	7/10	Good
Okamura [23]	1	1	0	1	0	0	1	1	1	1	1	7/10	Good
Yurt [17]	1	1	1	1	0	0	0	1	1	1	1	7/10	Good
Shih [21]	0	1	0	1	0	0	0	1	1	1	1	6/10	Good
Méndez [3]	1	1	1	1	1	0	1	0	1	1	1	8/10	Good

(Note: Scores: 1 = yes, 0 = no. PEDro criteria: 1, eligibility criteria; 2, random allocation; 3, concealed allocation; 4, baseline comparability; 5, blinded participants; 6, blinded therapists; 7, blinded assessors; 8, adequate follow-up; 9, intention-to-treat analysis; 10, between-group comparisons; 11, point estimates and variability).

## Data Availability

The data that support the findings of this study are available from the corresponding author, upon reasonable request.
